# Use of CRISPR/Cas9-mediated disruption of CNS cell type genes to profile transduction of AAV by neonatal intracerebroventricular delivery in mice

**DOI:** 10.1038/s41434-021-00223-3

**Published:** 2021-02-22

**Authors:** Tess Torregrosa, Sydney Lehman, Sam Hana, Galina Marsh, Shanqin Xu, Kathryn Koszka, Nicole Mastrangelo, Alexander McCampbell, Christopher E. Henderson, Shih-Ching Lo

**Affiliations:** Biogen, Cambridge, MA USA

**Keywords:** Genetic transduction, Gene therapy

## Abstract

Adeno-associated virus (AAV) transduction efficiency and tropism are conventionally determined by high expression of a fluorescent reporter gene. Emerging data has suggested that such conventional methods may underestimate AAV transduction for cells in which reporter expression from AAV vectors is undetectable. To explore an alternative method that captures AAV transduction in cells in which low expression of a cargo is sufficient for the intended activity, we sought after CRISPR/Cas9-mediated gene disruption. In this study, we use AAV to deliver CRISPR/guide RNA designed to abolish the genes NeuN, GFAP, or MOG expressed specifically in neurons, astrocytes, or oligodendrocytes respectively in the central nervous system (CNS) of mice. Abrogated expression of these cell-type-specific genes can be measured biochemically in CNS subregions and provides quantitative assessment of AAV transduction in these CNS cell types. By using this method, we compared CNS transduction of AAV9, AAV-PHP.B, and AAV-PHP.eB delivered via intracerebroventricular injection (ICV) in neonatal mice. We found both AAV-PHP.B and AAV-PHP.eB resulted in marked disruption of the NeuN gene by CRISPR/Cas9, significantly greater than AAV9 in several brain regions and spinal cord. In contrast, only modest disruption of the GFAP gene and the MOG gene was observed by all three AAV variants. Since the procedure of ICV circumvents the blood–brain barrier, our data suggests that, independent of their ability to cross the blood–brain barrier, AAV-PHP.B variants also exhibit remarkably improved neuronal transduction in the CNS. We anticipate this approach will facilitate profiling of AAV cellular tropism in murine CNS.

## Introduction

Recombinant adeno-associated viruses (AAVs) are commonly used to transfer exogenous DNA in vivo to the central nervous system (CNS) in mammals. The cargo DNA of AAV vectors can be designed to mediate editing in the host genome or drive transgene expression for studying the function of a gene in CNS [1, 2], modeling neurodegenerative diseases [3], or gene therapy treatments in animal models [4]. Broad CNS transduction requires optimal routes of administration of AAV for its delivery to different regions throughout the brain and spinal cord. In addition, researchers are racing to identify novel AAV serotypes with enhanced transduction efficiency and broad cellular tropism in the CNS, including neuronal and nonneuronal cell populations [4, 5]. Methods that facilitate transduction profiling are much needed for guiding AAV delivery optimization and novel capsid characterization.

Many research studies have utilized the AAV9 serotype to achieve broad transduction throughout the mouse CNS by intravenous (IV) injection [6–8] or neonatal intracerebroventricular (ICV) injection [9–11]. Further engineering of AAV9 variants has led to the discovery of AAV-PHP.B and AAV-PHP.eB with improved trans blood–brain barrier (BBB) capacity. These AAV-PHP.B variants showed enhanced cellular tropism and transduction efficiency throughout the CNS following IV injection in C57BL/6J mice [12–14]. However, comparisons between AAV9 and both AAV-PHP.B variants have not been examined following direct administration into the cerebral spinal fluid, which bypasses the BBB in mice. Mechanisms underlying highly efficient CNS transduction by AAV-PHP.B variants, other than transporting across the BBB, have not been explored [15–17].

AAV transduction patterns can be visualized at cellular levels by expression of a reporter gene or DNA barcode in the AAV vectors [11, 13, 14, 18–20] using immunohistochemistry (IHC) or in situ hybridization (ISH) though these conventional methods rely on high cargo expression levels. Relative AAV transduction efficiency can also be accurately measured in a higher throughput manner by quantitative polymerase chain reaction (qPCR) [12, 14, 18, 20, 21] or immunoblotting [22] analysis of cargo expression in tissues homogenates, albeit unable to probe cellular tropism. Recently, Lang et al. reported the use of CRISPR/guide RNA (sgRNA) delivered by AAV IV-injected in whole-body Cas9 knock-in mice to capture AAV transduction in cells in which low expression of sgRNA is sufficient for its gene editing activity [20]. In the transduced cells in peripheral tissues, CRISPR/sgRNA targets a stop cassette for removal, thereby enabling expression of the tdTomato reporter allele from the genomic locus. This CRISPR approach showed improved sensitivity in measuring AAV transduction of cells with both high and low levels of cargo expression. Furthermore, as sgRNA expression is driven by a ubiquitous pol III U6 promoter, this CRISPR approach also revealed transduction in cell types previously undetected by the reporter transgene expressed from a pol II promoter, which may be inactive in certain cell populations in vivo.

We have further expanded the idea of using AAV to deliver CRISPR/sgRNA in mice, and in turn assessing AAV transduction based on the outcome of CRISPR/sgRNA-mediated genome editing in vivo. We sought to develop an approach to assess AAV transduction in multiple cell types in the CNS by using CRISPR/sgRNA to disrupt cell-type-specific genes in the CNS. Platt et al. previously reported a CRISPR/sgRNA that targeted a neuronal-specific gene, NeuN (Rbfox3), for disruption. Following intraparenchymal injection in the brain, ~84% of the transduced cells had genome editing in both alleles, which intuitively corresponded to ~80% NeuN protein reduction in the bulk tissues at the injection site [23]. Here, we utilized a further improved CRISPR/sgRNA (termed sgNeuN) that targets the NeuN gene for disruption at 99.4% of biallelic editing rate (data not shown; Hana et al. [24] co-submitted for publication). By using neonatal ICV injection, AAV9, AAV-PHP.B and AAV-PHP.eB encoding sgNeuN were delivered into the whole-body Cas9 knock-in mice, backcrossed to C57BL/6J strain and confirmed to express Cas9 broadly throughout CNS. Neuronal transduction in brain subregions and spinal cord was assessed based on abrogated NeuN protein expression. To our surprise, despite that ICV injection circumvents the BBB, AAV-PHP.B variants showed superior neuronal transduction than AAV9 in murine CNS. In addition, we compared AAV9 and AAV-PHP.B variants for transduction in nonneuronal populations in the CNS by using a CRISPR/sgRNA that targets either an astrocyte-specific gene or an oligodendrocyte-specific gene for disruption.

## Materials and methods

### Animals

H11-Cas9 mice on C57BL/6J [B6J.129(Cg)-Igs2tm1.1(CAG-cas9*)Mmw/J; stock #028239; laboratory of M. Winslow, Stanford University, Stanford, CA] [25] constitutively expressing *Streptococcus pyogenes* Cas9 (Cas9) were purchased from Jackson Laboratory (Bar Harbor, ME). Homozygous H11-Cas9 mice were crossed to generate animals used in all experiments of this study. Mice within litters were randomized for the treatment groups. Experimenters were blinded to treatment. Animals were euthanized and excluded from tissue processing or further analysis if they exhibited hydrocephalus. All animal use and treatments were approved by the Biogen Institute Animal Care and Use Committee and followed the National Institute of Health *Guide for the Care and Use of Laboratory Animals*.

### sgRNA design

The sgRNA design for mouse NeuN (sgNeuN) (5′-GTTTGGGCTGCTGCTTCTCCG-3′) and for LacZ (sgLacZ) (5′-GTGCGAATACGCCCACGCGAT-3′) was previously described (Hana et al. co-submitted for publication). The sgRNA designs for mouse GFAP (sgGFAP), and mouse MOG (sgMOG) were designed in Benchling [26–28] based on specificity and efficiency scores. Seven GFAP and seven MOG sgRNA designs were screened in vitro with Cos1 and Neuro-2a cell lines for CRISPR efficiency (Supplementary Figs. 1, 2). The NeuN sgRNA, the most efficient GFAP sgRNA design #6 (5′-GAAGCCAGCATTGAGCGCCC-3′) and the most efficient MOG sgRNA design #4 (5′-GATGACAACTGGAGGAGAAGG-3′) were further used for AAV vector production. All sgRNAs contain a backbone structurally optimized for binding of Cas9 (5′-GTTTAAGAGCTATGCTGGAAACAGCATAGCAAGTTTAAATAAGGCTAGTCCGTTATCAACTTGAAAAAGTGGCACCGAGTCGGTGCTTTTTT-3′) [29].

### Plasmid design, vector production, and purification

GFAP and MOG overexpression vectors contain a CMV promoter that drives expression of mouse GFAP or mouse MOG fused with a Myc epitope tag. The Cas9 expression vector is a gift from Douglas Larigan (pVAX1-FLAG-NLS-SpCas9-NLS) which has a CMV promoter that drives expression of Cas9. Each AAV vector contains a pol III U6 promoter that drives expression of a sgRNA and a CBA promoter that drives expression of a green fluorescence protein (eGFP) fused with a nuclear localization signal (NLS). Single-stranded viral vectors were packaged into either AAV9, AAV-PHP.B, or AAV-PHP.eB at a titer of 5E13 vg/ml and were produced and purified (PackGene Biotech, Worcester, MA). Triple-plasmid transfection using polyethylenimine (PEI, Polyscience) was carried out to produce the recombinant AAV. The transfer plasmids AAV plasmid encoding the cargo described in the previous sgRNA design method section, pRep2CapX of AAV-X encoding Rep2 and CapX proteins plasmids, and pHelper were co-transfected into HEK293T cells. The cells were cultured in Dulbecco’s modified essential medium (DMEM; Invitrogen, USA) containing 10% fetal bovine serum (FBS) (Gibco, USA) and 1% penicillin–streptomycin antibiotics (Gibco, USA) at 37 °C. When the cells reached 80% confluence, they were transfected in 150 mm plates with 12 μg of pHelper plasmid, 10 μg of AAV pRep2CapX plasmids, and 6 μg of transfer AAV plasmids encoding the cargo for each plate. At 72 h post transfection, cells were harvested by 4000 × *g* centrifugation at 4 °C for 30 min. The pellet was collected and re-suspended in buffer containing 10 mM Tris-HCl, pH 8.0. The suspension was subjected to four freeze–thaw cycles by dry ice/ethanol and a 37 °C water bath. The cell debris was sonicated and then digested with DNase I (200 U in 1.5 ml) for 1 h at 37 °C. Following centrifugation at 10,000 × *g* for 10 min at 4 °C, the supernatant was collected as the AAV crude lysate. The crude lysate was diluted with 10 mM Tris-HCl pH 8.0 to a final volume of 10 ml and then bottom-loaded to a discontinuous gradient of 15, 25, 40, and 60% Iodixanol in a 39 ml ultracentrifuge tube (QuickSeal, 342414). After ultracentrifugation at 350,000 × *g* and 18 °C for 1 h, 3 ml fractions at lower position of 40% and 0.5 ml of 60% upper layer were collected. Ultracentrifugation was repeated at 350,000 × *g* and 18 °C for 1 h one more time and the fractions were desalted using a 100 kDa Cutoff Ultrafiltration tube (15 ml; Millipore, USA). The purified AAV were stored at −80 °C before usage. The viral titers were determined by SYBR Green qPCR.

### Neonatal intracerebroventricular (ICV) injections

On postnatal day 0 (P0), neonatal mice were cryo-anesthetized and then injected with AAV-sgNeuN/sgGFAP/sgMOG at either a low (5E10 vg) or high (20E10 vg) dose at a total volume of 4 µL. Mice injected with AAV-sgLacZ were only injected at a high dose. Viruses were diluted with phosphate-buffered saline (PBS) and mixed with Fastgreen dye (final concentration: 0.25%). Mock injected mice received an injection of 4 µL sterile PBS. The ICV injection procedure was adapted from Kim et al. [30]. ICV injections were performed perpendicular to the skull, located 1 mm lateral to the superior sagittal sinus, unilaterally between lambda and bregma, to a depth of 2 mm. Injections were performed with a 33-gauge, 10 µL, 45° bevel Hamilton syringe (Hamilton Company, Reno, NV, USA). Injection efficiency was monitored by the spread of the dye mixture throughout the lateral and the third ventricles. Afterwards, pups were placed inside of a warming chamber to recover body heat. Injected mice were weaned at 4 weeks and the tissues were harvested at 5 weeks for eGFP RNAscope, sgNeuN, and sgGFAP studies. Injected mice were weaned at 5 weeks and the tissues were harvested at 6 weeks for the sgMOG studies.

### In situ hybridization (ISH) RNAscope

**Fig. 1:**
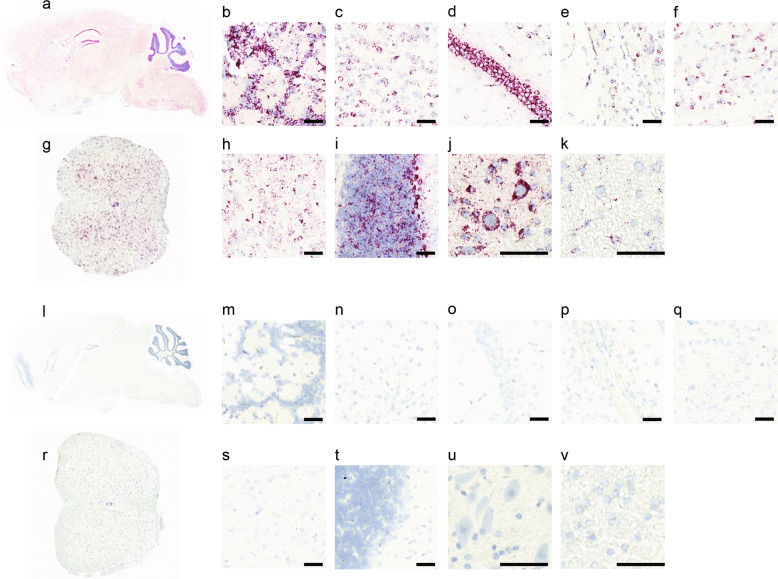
Cas9 mRNA expression in the CNS tissues in H11-Cas9 mice. Representative RNAscope images showed Cas9 mRNA expression, shown in violet, and cell nuclei, shown in blue, in various CNS regions in the Cas9 knock-in mice (**a**–**k**) and the age-matched wild-type mice (**l**–**v**): a sagittal section of the whole brain (**a** and **l**), olfactory bulb (**b** and **m**), cortex (**c** and **n**), hippocampus (**d** and **o**), corpus callosum (**e** and **p**), substantia nigra pars compacta (**f** and **q**), a full transverse section of the spinal cord (**g** and **r**), thalamus (**h** and **s**), cerebellum (**i** and **t**), gray matter area of the ventral spinal cord (**j** and **u**), and white matter area of the spinal cord (**k** and **v**). All scale bars are 50 µm.

**Fig. 2:**
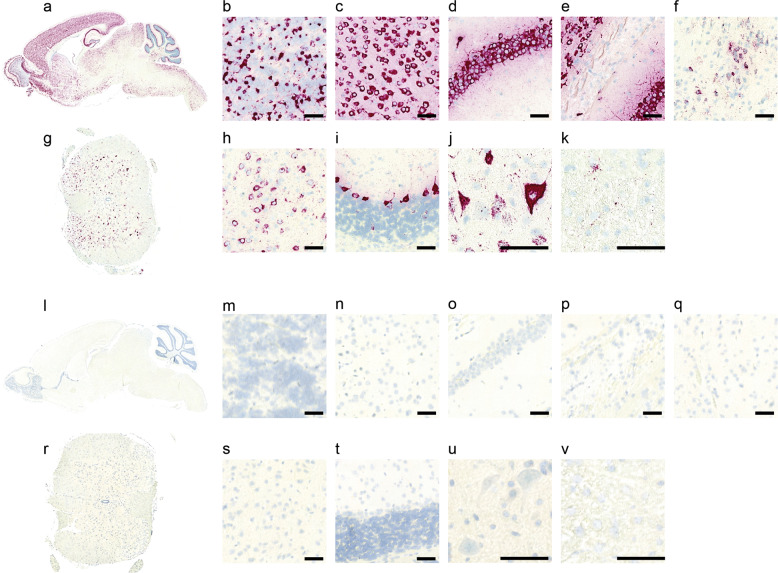
eGFP mRNA expression in the CNS tissues in C57BL/6J mice, following neonatal injection of AAV-PHP.eB-CBA-eGFP. Representative RNAscope images showed eGFP mRNA expression, shown in violet, and cell nuclei, shown in blue, in various CNS regions in C57BL/6J mice injected with AAV-PHP.eB (**a**–**k**) and PBS-injected control mice (**l**–**v**): a sagittal section of the whole brain (**a** and **l**), olfactory bulb (**b** and **m**), cortex (**c** and **n**), hippocampus (**d** and **o**), corpus callosum (**e** and **p**), substantia nigra pars compacta (**f** and **q**), a full transverse section of the spinal cord (**g** and **r**), thalamus (**h** and **s**), cerebellum (**i** and **t**), gray matter area of the ventral spinal cord (**j** and **u**), and white matter area of the spinal cord (**k** and **v**). All scale bars are 50 µm.

The whole brains and spinal cords were removed from mice. Samples were fixed in 10% neutral buffer formalin, processed, and embedded into paraffin. Brains were transected sagittally and embedded in the midline down orientation, and spinal cords were transected and embedded in the transverse orientation. ISH staining was performed on a Leica Biosystems’ BOND RX Autostainer (Leica Biosystems) using RNAscope^®^ 2.5 LSx Reagent Kit—RED (Advanced Cell Diagnostics) [31]. Staining was done according to the manufacturer’s instructions. Briefly, 5 µm thick sections were deparaffinized and rehydrated then subjected to target retrieval for 15 min at 95 °C using Leica Epitope Retrieval Buffer 2 (ER2) and protease treatment for 15 min at 40 °C, followed by specific probe-SpCas9 (ACDBio, 475788) hybridized to target RNA for the Cas9 homozygous and WT pups, and probe-eGFP (ACDBio, 400288) for the AAV-PHP.eB and mock transduced pups, respectively. The signal was amplified using a cascade of amplifier hybridizations to binding sites. Fast Red chromogenic detection was then performed. The background staining was assessed by negative control probe-dapB (ACDBio, 312038). Figures 1, 2 are from *n* = 3, biological replicates for Cas9 versus wild type and eGFP versus mock mice.

### Peggy Sue automated western blot

**Fig. 3:**
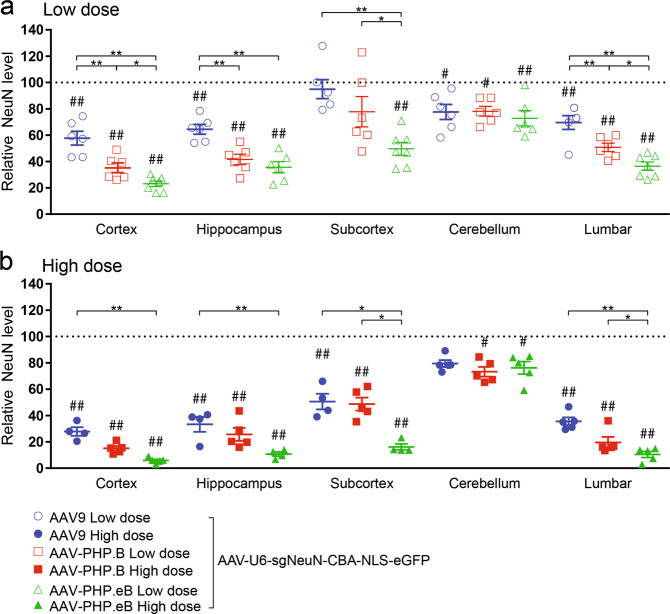
Robust yet varying degrees of NeuN protein reduction were shown across CNS regions following neonatal ICV injection of AAV-CRISPR/sgNeuN. Viruses (ssAAV-U6-sgNeuN-CBA-NLS-eGFP) were ICV injected at either a low (**a** 5E10 vg) or high dose (**b** 20E10 vg) in neonatal mice. After 5 weeks, multiple brain regions and spinal cord were dissected and the amount of the NeuN proteins remaining undisrupted in the bulk tissues was quantified using the Protein Simple Peggy Sue. For quantification of each AAV variant within each CNS region: the averages of the NeuN signals from AAV-CRISPR/sgLacZ (not shown) were normalized to 100% as baseline (dotted line), relative NeuN protein levels are presented as mean ± s.e.m., two-way ANOVA followed by Tukey’s test, between AAVs *p < 0.05; **p < 0.01, from baseline (sgLacZ) ^#^*p* < 0.05; ^##^*p* < 0.01.

**Fig. 4:**
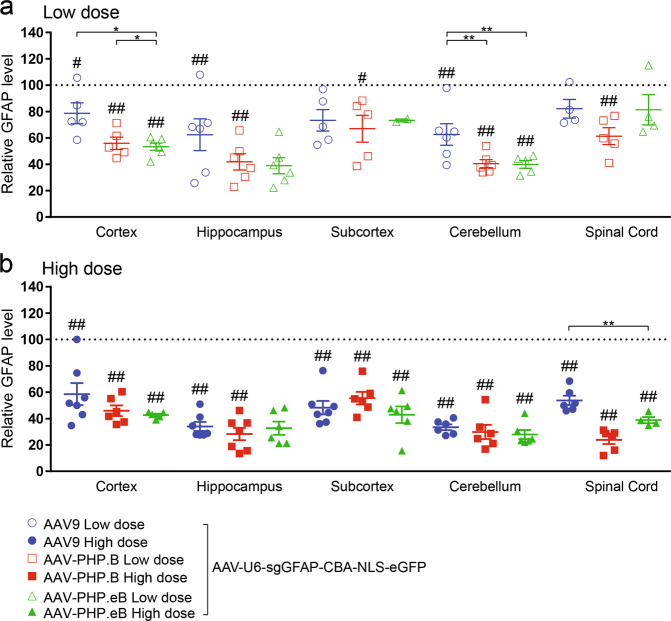
Modest and consistent GFAP protein reduction was shown across CNS regions following neonatal ICV injection of AAV-CRISPR/sgGFAP. Viruses (ssAAV-U6-sgGFAP-CBA-NLS-eGFP) were injected at either a low (**a** 5E10 vg) or high dose (**b** 20E10 vg) in neonatal mice. After 5 weeks, multiple brain regions and spinal cord were dissected and the amount of the GFAP proteins remaining undisrupted in the bulk tissues was quantified using the Protein Simple Peggy Sue. For quantification of each AAV variant within each CNS region: the averages of the GFAP signals from AAV-CRISPR/sgLacZ (not shown) were normalized to 100% as baseline (dotted line), relative GFAP protein levels are presented as mean ± s.e.m., two-way ANOVA followed by Tukey’s test, between AAVs *p < 0.05; **p < 0.01, from baseline (sgLacZ) ^#^*p* < 0.05; ^##^*p* < 0.01.

**Fig. 5:**
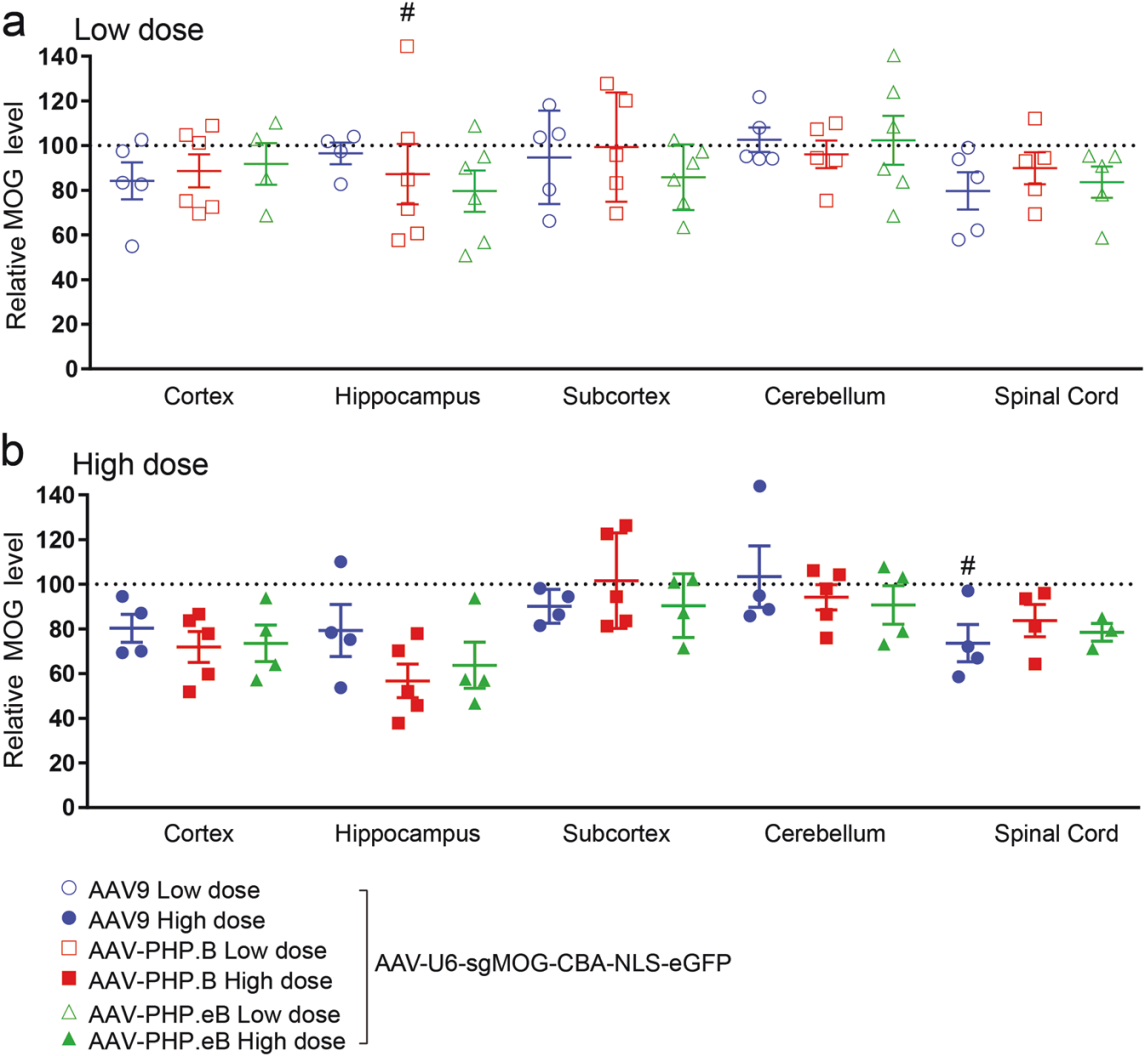
Minimal MOG protein reduction was observed across CNS regions following neonatal ICV injection of AAV-CRISPR/sgMOG. Viruses (ssAAV-U6-sgMOG-CBA-NLS-eGFP) were injected at either a low (**a** 5E10 vg) or high dose (**b** 20E10 vg) in neonatal mice. After 6 weeks, multiple brain regions and spinal cord were dissected and the amount of the MOG proteins remaining undisrupted in the bulk tissues was quantified using the Protein Simple Peggy Sue. For quantification of each AAV variant within each CNS region: the averages of the MOG signals from AAV-CRISPR/sgLacZ (not shown) were normalized to 100% as baseline (dotted line), relative MOG protein levels are presented as mean ± s.e.m., two-way ANOVA followed by Tukey’s test, from baseline (sgLacZ) ^#^*p* < 0.05.

The brain was sub-dissected into the cortex, hippocampus, subcortex, and cerebellum, and half of the spinal cord was collected. Tissues were homogenized in tissue lysis buffer [50 mM Tris-HCl (pH 7.5), 150 mM NaCl, 1% Triton X, 1% Na-deoxycholate (Sigma, D6750-106), 0.1% SDS, 8M Urea (Sigma, U4883), 5 mM EDTA, complete protease inhibitor (Roche, 04693124001), PhosSTOP phosphatase inhibitor (Roche, 4906845001), 1 mM phenylmethanesulfonylfluoride fluoride (Sigma, 93482), 1 mM dithiothreitol, 10 mM sodium fluoride, and 1 mM sodium orthovanadate (New England Biosciences, P07585)] by TissureLyser II (Qiagen). Briefly, tissues were placed in Safelock tubes (Eppendorf, 4036-3352) with a 5 mm stainless steel bead (Qiagen, 69989) and were homogenized twice with rapid agitation for 3 min each with a frequency of 30 Hz (TissueLyzer II). Homogenates were subsequently cleared via centrifugation at 4 °C, 375 × *g* for 20 min and the supernatant was collected. Lysates were electrophoresed through an automated Western Peggy Sue system with the 12–230 kDa detection module according to manufacturer’s instructions (Protein Simple, SM-S001) [32]. Lysates were diluted to the linear dynamic range in 0.1× Sample buffer (Protein Simple, San Jose, CA, 042-195). For NeuN protein analysis, lysates were incubated with 1:250 of anti-NeuN antibody (CST, clone D3S3I, 12943) and either 1:100 anti-GAPDH antibody (CST, clone 14C10, 2118) or 1:500 anti-B-actin (CST, clone 8H10D10, 3700). For GFAP protein analysis, samples were incubated with 1:100 anti-GFAP (CST, clone GA5, 3670) and 1:100 anti-GAPDH. For MOG protein analysis, samples were incubated with 1:100 anti-MOG (CST, clone E5K6T, 96457) and 1:100 anti-GAPDH. Anti-mouse detection module (Protein Simple, DM-002) and anti-rabbit detection module (Protein Simple, DM-001) were used following incubation with corresponding primary antibodies. Figures 3–5 are from *n* = 2–7, biological replicates for mice injected with low and high doses of AAV9, AAV-PHP.B, and AAV-PHP.eB.

### In vitro sgRNA screen in Cos1 cell line

The sgRNAs were compared for their efficiency in facilitating CRISPR-mediated disruption of the target gene expressed from a co-transfected plasmid in Cos1 cells (ATCC, Manassas, VA, CAT#CRL-1650). Cos1 cells were maintained in DMEM supplemented with 10% heat-inactivated FBS, 1% penicillin–streptomycin (Gibco, 15070-063), and 2 mM L-glutamine. FuGENE HD (Promega, San Luis Obispo, CA, E2311) was used for transfection according to the manufacturer’s instructions. Briefly, the GFAP or MOG overexpression plasmid, Cas9 expression plasmid, and sgRNA plasmid were mixed in a 1:4:5 ratio to a total of 0.5 µg, and incubated with 1.5 µL of FuGENE HD in 25 µL Opti-MEM (Thermofisher, 31985962) prior to adding to the cultured cells. Twenty-four hours after transfection, cell lysates were harvested in 200 µL Novex Tris-Glycine SDS Sample Buffer (1.6×, Thermofisher, LC2676) with NuPage Sample Reducing Agent (Thermofisher, NP0009) and heated at 70 °C for 5 min (ThermoMixer C, Eppendorf). Lysates were sonicated at a force of 4 for 20 strokes (60 Sonic Dismembrator, Fisher Scientific). The lysates were subjected to western blotting analysis.

### In vitro sgRNA screen in Neuro-2a cell line

Neuro-2a cell line with tetracycline-inducible Cas9 expression, (SL508, GeneCopoeia, Rockville, MD), was also used to examine sgRNAs for their efficiency in facilitating CRISPR-mediated disruption of the target gene expressed from a co-transfected plasmid. Neuro-2a cells were maintained in DMEM supplemented with 10% heat-inactivated FBS, 1% penicillin–streptomycin (Gibco, 15070-063), and 2 mM L-glutamine. Cells were treated with 0.01, 0.1, or 1 µg/ml of doxycycline to induce expression of Cas9. After 24 h, cells were co-transfected with the GFAP or MOG overexpression plasmid and sgRNA plasmid in a 1:9 ratio by FuGENE (Promega, San Luis Obispo, CA, E2311) according to the manufacturer’s instructions. 24 h after transfection, cell lysates were harvested in 200 µL Novex Tris-Glycine SDS Sample Buffer (1.6×, Thermofisher, LC2676) with NuPage Sample Reducing Agent (Thermofisher, NP0009) and heated at 70 °C for 5 min (ThermoMixer C, Eppendorf). Lysates were sonicated at a force of 4 for 20 strokes (60 Sonic Dismembrator, Fisher Scientific). The lysates were subjected to Western blotting analysis.

### Western blotting and antibodies

Lysates were electrophoresed through NuPage 4–12% Bis-Tris Midi Gels (Thermofisher, WG1403BX10) and transferred to nitrocellulose membranes in Trans-blot Turbo Transfer Pack (Bio-Rad, 1704159) using Trans-blot Turbo transfer system (Bio-Rad). The blocking of membranes and subsequent antibody incubations were performed by using Odyssey blocking buffer (LI-COR Biosciences, 927-50000) according to the manufacturer’s instructions. Primary antibodies against Myc (1:2000, Enzo BML-SA294-0500), b-tubulin (1:5000, Li-Cor, 926-422-11), b-actin (1:10,000, Li-Cor, 926-422-10) and FLAG (F1804, Millipore Sigma) were purchased from commercial sources. The IRDye 800CW-conjugated donkey anti-mouse (Li-Cor, 925-32212) and IRDye 680CW-conjugated donkey anti-rabbit (Li-Cor, 925-68073) secondary antibodies were obtained from Li-Cor Biosciences. Immunoblot signals were visualized by the Odyssey CLx infrared imaging and quantified by Li-Cor Odyssey application software.

### Statistical analysis

In Figs. 3–5, quantification was performed for each AAV variant within each CNS region. The averages of the protein signals from AAV-CRISPR/sgLacZ (not shown) were normalized to 100% as baseline (dotted line). Remaining protein levels after CRISPR-mediated protein disruption are presented as means ± standard error of the mean (s.e.m.). Statistical analyses (a two-way ANOVA followed by a Tukey’s test) were performed using GraphPad Prism 7 software. Experimental groups were considered to be significant for *p* values < 0.05 and <0.01 for all experiments. The *p* value was only corrected within each CNS region. Protein levels significantly different between AAV variants are denoted with a “*” sign. Within each AAV variant, protein levels significantly different from the corresponding baseline (sgLacZ) are denoted with a “#” sign.

## Results

### H11-Cas9 mice have broad Cas9 expression in the CNS

Robust Cas9 mRNA signal was detected throughout the CNS in H11-Cas9 knock-in mice via RNAscope by violet dye-conjugated probe with complementary sequences to Cas9 mRNA (Fig. 1a–k). In contrast, such signal was nearly undetectable in age-matched wild-type mice, indicating good specificity of the probe (Fig. 1i–v). Overall, there was widespread Cas9 expression throughout the brain (Fig. 1a). Upon further inspection, multiple brain regions stained positive for Cas9 mRNA expression, predominantly in cytoplasm of the cell bodies, including cortex, regions of the subcortex, hippocampus, substantia nigra pars compacta, thalamus, striatum, and cerebellum (Fig. 1b–f, h, i). The spinal cord was also stained positive for Cas9 expression in cell bodies in both the gray matter and the white matter (Fig. 1g, j, k). Overall, Cas9 staining intensity per cell varies and was observed to be lower in the white matter of the spinal cord (Fig. 1j) than in the gray matter of the spinal cord (Fig. 1k). This data suggests H11-Cas9 knock-in mice display broad Cas9 expression in the brain and the spinal cord, despite variable expression level from cell to cell.

### RNAscope confirms transduction throughout CNS but lack of cerebellar granule cell transduction in the brain

The lateral ventricles of neonatal mice of C57BL/6J strain were ICV injected with AAV-PHP.eB on postnatal day 0 (P0). The AAV-PHP.eB vector contains the CBA promoter to drive broad expression of eGFP. AAV transduction of AAV-PHP.eB was assessed by RNAscope using a violet dye-conjugated probe with complementary sequences to eGFP mRNA. Positive staining for eGFP mRNA was detected in many of the CNS regions (Fig. 2), indicating broad transduction of AAV-PHP.eB throughout the brain and the spinal cord (Fig. 2a–k). In particular, olfactory bulb, cortex, hippocampus, corpus callosum, substantia nigra pars compacta, and thalamus displayed high levels of eGFP mRNA expression (Fig. 2b–f, h). In the cerebellum region, Purkinje cells displayed high intensity of eGFP staining signal, whereas cell bodies in the granule cell layer were stained negative for eGFP mRNA (Fig. 2i). Finally, the spinal cord displayed high eGFP staining signal in the gray matter (Fig. 2j) and modest yet detectable eGFP staining signal in the white matter (Fig. 2k). This data suggests, following neonatal ICV injection, AAV-PHP.eB can lead to broad transduction in CNS in C57BL/6J mice, except in the granule cells of cerebellum.

### CRISPR-mediated disruption of the NeuN gene for assessing neuronal transduction of AAV in CNS

To evaluate AAV transduction in neurons, NeuN (Rbfox3), a commonly used neuronal-specific marker gene [33], was selected as a target gene for CRISPR-mediated gene disruption, following administration of AAV that delivers sgRNA to the transduced cells. Whole-body NeuN knockout mice develop normally and exhibit unchanged cell viability, normal brain morphology, and unaffected locomotion at 7–11 weeks of age [34], indicating preservation of cell viability following abrogated NeuN expression. P0 neonatal H11-Cas9 mice were ICV injected with either AAV9, AAV-PHP.B or AAV-PHP.eB. The AAV vectors contain the U6 promoter to drive broad expression of CRISPR/sgRNA that targets the NeuN gene for disruption (termed sgNeuN, Hana et al. co-submitted). Neuronal transduction efficiency of AAV was inferred by measuring NeuN protein expression remained in the neurons lacking CRISPR/sgNeuN activity. We examined the cortex, hippocampus, subcortex, cerebellum, and lumbar regions using an anti-NeuN Protein Simple Peggy Sue assay, an automated immunoblotting analysis (Fig. 3; Supplementary Fig. S3a).

AAV9, AAV-PHP.B, and AAV-PHP.eB transduction resulted in a significant reduction of NeuN protein levels in multiple CNS regions (*p* < 0.01), when compared to the baseline of NeuN protein levels in the H11-Cas9 mice ICV injected with AAVs encoding for a non-specific CRISPR/sgRNA that targets the LacZ gene (β-galactosidase) for disruption (termed sgLacZ). Robust reduction of NeuN was observed in the cortex, hippocampus, and lumbar regions, ranging from 30.4 ± 5.2% (lumbar) to 76.9 ± 2.1% (cortex) for the low dose, and from 66.6 ± 3.0% (lumbar) to 94.9 ± 1.0% (cortex) for the high dose. For the subcortex region, when administered at the low dose, only AAV-PHP.eB transduction led to a significantly reduction of NeuN at 52.3 ± 4.9%, while transduction of all three AAVs led to more prominent reductions of NeuN protein levels at the high dose, ranging from 50.2 ± 5.9% to 84.5 ± 2.3% (*p* < 0.01). In the cerebellum region, regardless of the dose, all three AAVs resulted in a very modest reduction of NeuN at ~24%, indicating poor neuronal transduction in the cerebellum. Nevertheless, when administered at the low dose, transduction of both AAV-PHP.B and AAV-PHP.eB resulted in significantly greater reduction of NeuN than AAV9 throughout the cortex, hippocampus, subcortex, and lumbar regions (*p* < 0.01). Taken together, these results suggest that at a high dose, AAV9 and AAV-PHP.B variants broadly and efficiently transduce neurons in the cortex, hippocampus, subcortex, and lumbar CNS regions after P0 ICV delivery. In addition, AAV-PHP.B variants have the higher neuronal transduction efficiency than AAV9, except in the cerebellum.

### CRISPR-mediated disruption of the GFAP gene for assessing astrocyte transduction of AAV in CNS

To evaluate AAV transduction in astrocyte, we selected GFAP, the leading, well characterized mature astrocyte marker gene [35], for CRISPR-mediated gene disruption, following administration of AAV that delivers sgRNA to the transduced cells. In GFAP knockout mice, astrocytes were viable and showed unaffected proliferation [36], and the mouse exhibited normal motor behavior [37], indicating preservation of cell viability following abrogated GFAP expression. To identify CRISPR/sgRNA that potently disrupts GFAP expression, seven GFAP-targeting sgRNAs (termed sgGFAP) were assessed for their CRISPR activity in two cell lines. Cos-1 cells were co-transfected with expression vectors for mouse GFAP, Cas9 and sgGFAP. Seven sgGFAPs were compared for their ability to facilitate reduction of overexpressed mouse GFAP proteins by immunoblotting analysis (Supplementary Fig. S1a, b). In addition, engineered Neuro-2a cells were induced to express Cas9 at varying levels, and were subsequently co-transfected with expression vectors for mouse GFAP and sgGFAP. The sgGFAP #6 was found to consistently result in marked reduction of overexpressed mouse GFAP proteins in both cell lines, even when Cas9 was induced to express at minimal levels (Supplementary Fig. S1c, d).

Next, P0 neonatal H11-Cas9 mice were ICV injected with either AAV9, AAV-PHP.B or AAV-PHP.eB containing the U6 promoter to drive broad expression of CRISPR/sgGFAP #6. AAV transduction in astrocytes was assessed by measuring reduction of endogenous GFAP proteins in the cortex, hippocampus, subcortex, cerebellum, and lumbar regions using an anti-GFAP Protein Simple Peggy Sue assay (Fig. 4; Supplementary Fig. S3b).

When administered at low dose (Fig. 4a), transduction of AAV9-sgGFAP #6 resulted in significant reduction of GFAP protein levels in the cortex (21.4 ± 7.9%), hippocampus (37.6 ± 12.1%), and cerebellum (37.4 ± 8.2%). Transduction of AAV-PHP.B-sgGFAP #6 led to significant reduction of GFAP protein levels in all CNS regions, ranging from 33.0 ± 10.2% (subcortex) to 59.5 ± 3.0% (cerebellum) (*p* < 0.05). Transduction of AAV-PHP.eB-sgGFAP #6 resulted in significant reduction of GFAP protein levels in the cortex (46.7 ± 2.9%) and cerebellum (60.2 ± 2.9%). When administered at high dose (Fig. 4b), transduction of all three AAVs resulted in marked reduction of GFAP across almost all CNS regions examined, ranging from 41.4 ± 8.3% (cortex) to 76.1 ± 3.2% (lumbar). Comparable degree of GFAP reduction was caused by transduction of AAV9, AAV-PHP.B and AAV-PHP.eB, indicating similar astrocyte transduction by all three AAVs. These results suggest that AAV9 and AAV-PHP.B variants are able to broadly transduce astrocytes throughout the CNS with modest efficiency, following P0 ICV injection.

### CRISPR-mediated disruption of the MOG gene for assessing oligodendrocyte transduction of AAV in CNS

To evaluate AAV transduction in oligodendrocytes MOG, a mature oligodendrocyte-specific marker [38] was selected for CRISPR-mediated gene disruption, following administration of AAV that delivers CRISPR to the transduced cells. MOG knockout mice develop normally, have indistinguishable myelin sheaths compared to wild-type mice, and exhibit normal motor behavior for up to 20 months of age [39]. Unlike MOG, other oligodendrocyte-specific markers such as myelin basic protein [40] and Olig2 [41] knockout mice result in mortality phenotypes. To identify CRISPR/sgRNA that potently disrupts MOG expression, seven MOG-targeting sgRNAs (termed sgMOG) were assessed for their CRISPR activity in two cell lines. Cos-1 cells were co-transfected with expression vectors for mouse MOG, Cas9 and sgMOG. Seven sgMOGs were compared for their ability to facilitate reduction of overexpressed mouse MOG proteins by immunoblotting analysis. In addition, engineered Neuro-2a cells were induced to express Cas9 at varying levels, and were subsequently co-transfected with expression vectors for mouse MOG and sgMOG. In Cos-1 cells, several sgMOG designs resulted in marked reduction of overexpressed mouse MOG proteins (Supplementary Fig. S2a, b) and in Neuro-2a cells, sgMOG #4 was found to result in marked reduction of overexpressed mouse MOG proteins even upon low expression levels of Cas9 (Supplementary Fig. S2c, d).

Next, P0 neonatal H11-Cas9 mice were ICV injected with either AAV9, AAV-PHP.B or AAV-PHP.eB containing the U6 promoter to drive broad expression of CRISPR/sgMOG #4. Oligodendrocyte transduction was evaluated by measuring reduction of endogenous MOG proteins in the cortex, hippocampus, subcortex, cerebellum, and lumbar regions using an anti-MOG Protein Simple Peggy Sue assay (Fig. 5; Supplementary Fig. S3c). Regardless of the AAV serotypes used or the doses of AAV administered, we observed a slight reduction of the MOG proteins of ~10–20% by CRISPR/sgMOG #4 across almost all CNS subregions examined, albeit not statistically significant. This result suggests poor oligodendrocyte transduction by AAV9 and AAV-PHP.B variants in the brain and the spinal cord tissues after P0 ICV delivery.

## Discussion

In this study, we demonstrated the use of a CRISPR/sgRNA gene editing approach to quantitatively compare AAV9, AAV-PHP.B, and AAV-PHP.eB transduction efficiency and cellular tropism in the mouse CNS. Here we report the first comparison of these AAVs when delivered by ICV injection in neonatal C57BL/6J. The CRISPR/sgRNAs were designed to target CNS cell-type specific genes for disruption, including mouse NeuN gene [33] expressed in neurons, mouse GFAP gene expressed in astrocytes [35], and mouse MOG gene expressed in oligodendrocytes [42]. Following CRISPR-mediated gene disruption in the transduced cells, we infer AAV transduction efficiency in these cell types by measuring abrogated expression of NeuN, GFAP, and MOG respectively in multiple subregions dissected from the brain and spinal cord tissues. Peggy Sue, an automated western blotting system, was used to facilitate higher throughput analysis of the remaining NeuN, GFAP and MOG proteins expressed from undisrupted alleles in the non-transduced cells. Overall, we observed robust disruption of the NeuN gene throughout CNS, except for cerebellum, indicating subregional-dependent neuronal transduction of AAV-PHP.B variants, following neonatal ICV delivery.

RNAscope [31], a cutting-edge ISH method with single-molecule sensitivity, has the advantage for its ability to limit the bias detection associated with errors derived from processing tissues and microscope handling, commonly encountered by IHC [43]. By using RNAscope, we first confirmed widespread expression of Cas9 in the brain and the spinal cord tissues in the H11-Cas9 mice, thereby validating the use of H11-Cas9 mice for CNS gene editing upon delivery of sgRNA. Subsequently, we used RNAscope to visualize the reporter gene eGFP expression from AAV-PHP.eB. Consistent with the Peggy Sue results showing marked disruption of NeuN by AAV-PHP.eB-sgNeuN in multiple regions in the brain and the spinal cord (Fig. 3), we observed broad eGFP expression throughout the CNS (Fig. 2). Furthermore, we qualitatively confirmed lack of transduction of granule cells in the cerebellum by using RNAscope (Fig. 2), in agreement with the results from the CRISPR/sgRNA approach (Fig. 3). The rapidly growing AAV toolbox including variants such as AAV-PHP.B4 and AAV-PHP.N [14] needs a high throughput method to guide efforts in AAV delivery optimization and novel capsid characterization. We believe, in complementary to the conventional methods based on imaging of the reporter gene expression, we provided a CRISPR/sgRNA strategy to facilitate profiling of AAV transduction in CNS.

The idea of using CRISPR/sgRNA for AAV profiling in vivo can be further expanded to increase the versatility of the toolkit. For example, it may be applied to other tissues such as the peripheral nervous system, eye, heart, or skeletal muscle to assess transduction of AAV capsids designed to target these tissues via different routes of administration [14]. In addition, alternative to relying on whole-body Cas9 knock-in mice, one can use a CRISPR/sgRNA approach directly in wild-type animals by packaging a small Cas9 ortholog, along with a sgRNA in the same AAV vector [44, 45]. In particular, a CRISPR/sgRNA approach that enables profiling of novel engineered AAVs in non-human primates (NHP) will facilitate translational research in gene therapy [14, 46, 47]. The sgRNA that targets the NHP gene for NeuN, GFAP, or MOG will need to be redesigned and co-delivered with the Cas nuclease. Furthermore, the antibodies that cross react with the NHP NeuN, GFAP, and MOG proteins raised against these NHP proteins should be used.

AAV delivery of other CRISPR/sgRNA systems may have translational values for gene therapy. For example, CRISPR-mediated precise gene editing via homology directed repair and CRISPR activation (CRISPRa) may be used to re-express the wild-type form of a gene or to upregulate an otherwise deactivated gene [48]. For successful translation of this toolkit for gene therapy applications different properties should be carefully examined. First, age can have an impact on the AAV transduction efficiency, and this strategy must be next assessed in in adult mice [49, 50]. Furthermore, different routes of injection have also been shown to influence transduction and cargo expression for many AAV serotypes, and therefore it is essential to choose an optimal route for CNS gene therapies [7, 47, 51]. Finally, additional experiments must be performed in multiple mouse strains. Notably, AAV-PHP variants have been shown to efficiently transduce cells in the CNS of C57BL/6 mouse strain, but resulted in limited transduction in the CNS of BALB/cJ and FVB/NJ strains following IV delivery [14–17].

Unlike the robust disruption of the NeuN gene upon high neuronal transduction by AAV-PHP.B-sgNeuN and AAV-PHP.eB-sgNeuN, we observed modest disruption of the GFAP gene throughout CNS (Fig. 4), and poor disruption of the MOG gene (Fig. 5), indicating a bias of AAV-PHP.B variants toward transducing neurons over astrocytes or oligodendrocytes. Such limited transduction of astrocytes and oligodendrocytes could be due to the age of the animal at the time of injection. Indeed, neuronal biased transduction of AAV9 has been reported when AAV is ICV injected in neonatal mice on P0, while astrocyte biased transduction was observed when ICV injection of AAV9 was performed on postnatal day 3 (P3) [9]. Studies of astrocyte proliferation and migration in neonatal brain development demonstrated that the first wave of astrogliogenesis continues to peak until P3, and the second wave of astrogliogenesis can extend into postnatal week 3 [52–54]. It is interesting to postulate that the viral particles ICV injected at P0 may not possess long lasting infectivity for 3 weeks in vivo to transduce the astrocyte population developed later in the neonatal brain. Lastly, it is worth noting that when intravenously delivered in the adult C57BL/6J mice, AAV-PHP.B and AAV-PHP.eB also showed greater transduction in neurons than in oligodendrocytes [12–14, 22], consistent with our results.

AAV-PHP.B and AAV-PHP.eB were identified in a directed evolution process, in which vectors from a library of modified AAV9 capsids with additional seven-amino-acid insert were recovered for enhanced CNS tropism after systemic IV delivery [12–14]. Although the mechanisms underlying AAV transduction in vivo are not well understood and involve multiple intermediate steps, it was hypothesized that these two AAV-PHP.B variants have distinct properties which allow for brain vascular association or BBB transcytosis [13]. Subsequent studies from Wilson lab, Deverman lab, and Sena-Esteves lab independently identified lymphocyte antigen 6 complex (LY6A) as a receptor for AAV-PHP.B and AAV-PHP.eB and binds to their seven-amino-acid insert with high binding affinity [15–17]. LY6A is a glycosylphosphatidylinositol (GPI)-anchored protein. It is expressed on the surface of endothelial cells, a key cell type that forms the BBB. Disruption of LY6A resulted in reduced transduction of mouse brain endothelial cells by AAV-PHP.eB [15]. Interestingly, C57BL/6J animals, a mouse strain exhibiting CNS permissiveness to IV-injected AAV-PHP.B, have high levels of LY6A expression in the microvascular endothelial cells. In contrast, LY6A expression is substantially reduced in BALB/cJ mouse strain [16], in which the CNS is impermeable to AAV-PHP.B delivered systemically. Consistently, Sena-Esteves lab recently showed that ICV injection of AAV-PHP.B in adult mice resulted in widespread transduction throughout the brain in C57BL/6J mice, while restricted AAV transduction was seen in BALB/cJ mice, further hinting at mechanisms, other than BBB transcytosis, responsible for CNS transduction of AAV-PHP.B [16]. Additional mechanistic studies will be needed to elucidate BBB-independent pathways that can be explored to further evolve or engineer AAV-PHP.B capsids.

## Supplementary information

Supplementary Figure Legends
