# Supplementary material for: Use of CRISPR/Cas9-mediated disruption of CNS cell type genes to profile transduction of AAV by neonatal intracerebroventricular delivery in mice
Source: Gene Ther. 2021 Feb 22;28(7-8):456–68. doi: 10.1038/s41434-021-00223-3 (PMC8376643; doi:10.1038/s41434-021-00223-3)
Supplement: Supplementary file 1 — Supplementary Figure Legends [file 41434_2021_223_MOESM1_ESM.docx]

**Supplementary Figure Legends**

Figure S1. *In vitro* validation of GFAP targeting sgRNA design #6 consistently reduces overexpression of GFAP in Cos1 and Neuro-2a cell lines. Seven GFAP-targeting sgRNAs were co-transfected with GFAP and Cas9 expression plasmids in Cos1 cells (a and b). Seven GFAP-targeting sgRNAs were co-transfected with the GFAP expression plasmid in Neuro-2a cells (c and d). Neuro-2a cells were induced to express Cas9 with 1, 0.1, or 0.01 µg/mL doxycycline. After 24 hours remaining GFAP proteins remaining undisrupted was quantified with western blot analysis.

Figure S2. *In vitro* validation of MOG targeting sgRNA design #4 reduces overexpression of MOG in Cos1 and Neuro-2a cell lines. Seven MOG-targeting sgRNAs were co-transfected with MOG and Cas9 expression plasmids in Cos1 cells (a and b). Seven MOG-targeting sgRNAs were co-transfected with the MOG expression plasmid in Neuro-2a cells (c and d). Neuro-2a cells were induced to express Cas9 with 1, 0.1, or 0.01 µg/mL doxycycline. After 24 hours remaining MOG proteins remaining undisrupted was quantified with western blot analysis.

Figure S3. Representative images showed simulated Western blot from Peggy Sue for *in vivo* protein analysis of NeuN (a), GFAP (b), and MOG (c) in the cortex. Neonatal mice were injected with low (L) and high (H) doses of AAV9, AAV-PHP.B, and AAV-PHP.eB vectors encoding for sgLacZ, sgNeuN, sgGFAP, or sgMOG. After 5-6 weeks, multiple brain regions and spinal cord were dissected and the amount of the NeuN (a), GFAP (b) and MOG (c) proteins remaining undisrupted in the bulk tissues was analyzed using the Protein Simple Peggy Sue.
